# MiR-486-5p negatively regulates oncogenic NEK2 in hepatocellular carcinoma

**DOI:** 10.18632/oncotarget.17635

**Published:** 2017-05-05

**Authors:** Shun-Jun Fu, Jian Chen, Fei Ji, Wei-Qiang Ju, Qiang Zhao, Mao-Gen Chen, Zhi-Yong Guo, Lin-Wei Wu, Yi Ma, Dong-Ping Wang, Xiao-Feng Zhu, Xiao-Shun He

**Affiliations:** ^1^ Organ Transplant Center, The First Affiliated Hospital, Sun Yat-sen University, Guangzhou 510080, P. R. China; ^2^ Guangdong Provincial Key Laboratory of Organ Donation and Transplant Immunology, The First Affiliated Hospital, Sun Yat-sen University, Guangzhou 510080, P. R. China; ^3^ Guangdong Provincial International Cooperation Base of Science and Technology (Organ Transplantation), The First Affiliated Hospital, Sun Yat-sen University, Guangzhou 510080, P. R. China

**Keywords:** NEK2, MiR-486-5p, hepatocellular carcinoma, tumor progression, prognosis

## Abstract

NEK2 is a member of the NIMA-related family of serine/threonine centrosomal kinases. We analyzed the relationship between differential expression of NEK2 and hepatocellular carcinoma (HCC) patient outcomes after liver transplants. We also studied the microRNAs that affect NEK2 expression. Analysis of multiple microarrays in the Oncomine database revealed that NEK2 expression was higher in HCC tissues than adjacent normal liver tissues. High NEK2 expression correlated with tumor size, pathological grade and macro- and microvascular invasion. Consequently, patients exhibiting high NEK2 expression had poorer prognosis. This was corroborated by our multivariate analysis that showed NEK2 to be an independent prognostic factor for HCC patient survival. Further, high NEK2 expression promoted proliferation, colony formation, migration and invasion of HCC cell lines. Tumor xenograft data from Balb/c nude mice demonstrated that HCC cells with high NEK2 expression formed larger tumors than those with low NEK2 expression. Finally, we showed that miR-486-5p suppressed NEK2 by directly binding to its transcript 3′UTR. We also demonstrated an inverse relationship between miR-486-5p and NEK2 expression in HCC patients. These findings suggest miR-486-5p negatively regulates NEK2, which is a critical prognostic indicator of HCC patient survival after liver transplantation.

## INTRODUCTION

Hepatocellular carcinoma (HCC) is the fifth most common cancer and the third leading cause of cancer-related death worldwide, with more than 780,000 new cases and around 745,000 deaths each year [1]. The incidence of HCC is increasing worldwide, especially in the United States and Europe [2, 3]. China accounts for nearly 55% of all HCC cases in the world [4]. Hepatic resection and liver transplantation are the standard radical treatments for patients with HCC. However, the 5 year survival rate of 30% is very poor inspite of these radical treatments [1]. The main risk factors affecting long term survival of HCC patients is high rate of recurrence and metastasis. Therefore it is critical to understand the underlying mechanisms of HCC metastasis in order to find novel therapeutic targets that would improve survival rates of HCC patients.

One potential therapeutic target is NEK2 [NIMA (never in mitosis gene A)-related expressed kinase 2], which is a member of the NIMA family of serine/threonine centrosomal kinases that play a vital role in regulating cell cycle and mitosis during cell division [5, 6]. Enhanced NEK2 levels lead to chromosomal instability (CIN) including centrosomal abnormalities that lead to aberrant amplification of chromosomes and chromosomal rearrangements [7–11]. Overexpression of NEK2 is observed in a number of cancers including breast cancer, pancreatic cancer, lung adenocarcinoma, cholangiocarcinoma, colorectal cancer (CRC) and non-Hodgkin lymphoma [12–18]. Meanwhile, two studies indicated that overexpression of NEK2 is a strong biomarker for drug resistance and poor prognosis in multiple myeloma [8, 19]. These findings suggested that NEK2 is a critical oncogene and could be a potential anti-cancer therapeutic target.

NEK2 has also been associated with HCC. Recently, NEK2 levels were found to influence the biological behaviors of HCC cells and its high expression was found to indicate poor prognosis in HCC patients after hepatectomy [20–22]. Since the role of NEK2 and its regulation has not been fully understood in context of HCC, we investigated the consequences of high or low NEK2 expression in HCC patient samples and HCC cell lines to understand the prognostic relevance of NEK2 in HCC. Also, we investigated the miRNAs that regulate NEK2 expression and its clinical relevance to HCC patients that underwent liver transplantation.

## RESULTS

### NEK2 is overexpressed in human HCC tissues and cell lines

**Figure 1:**
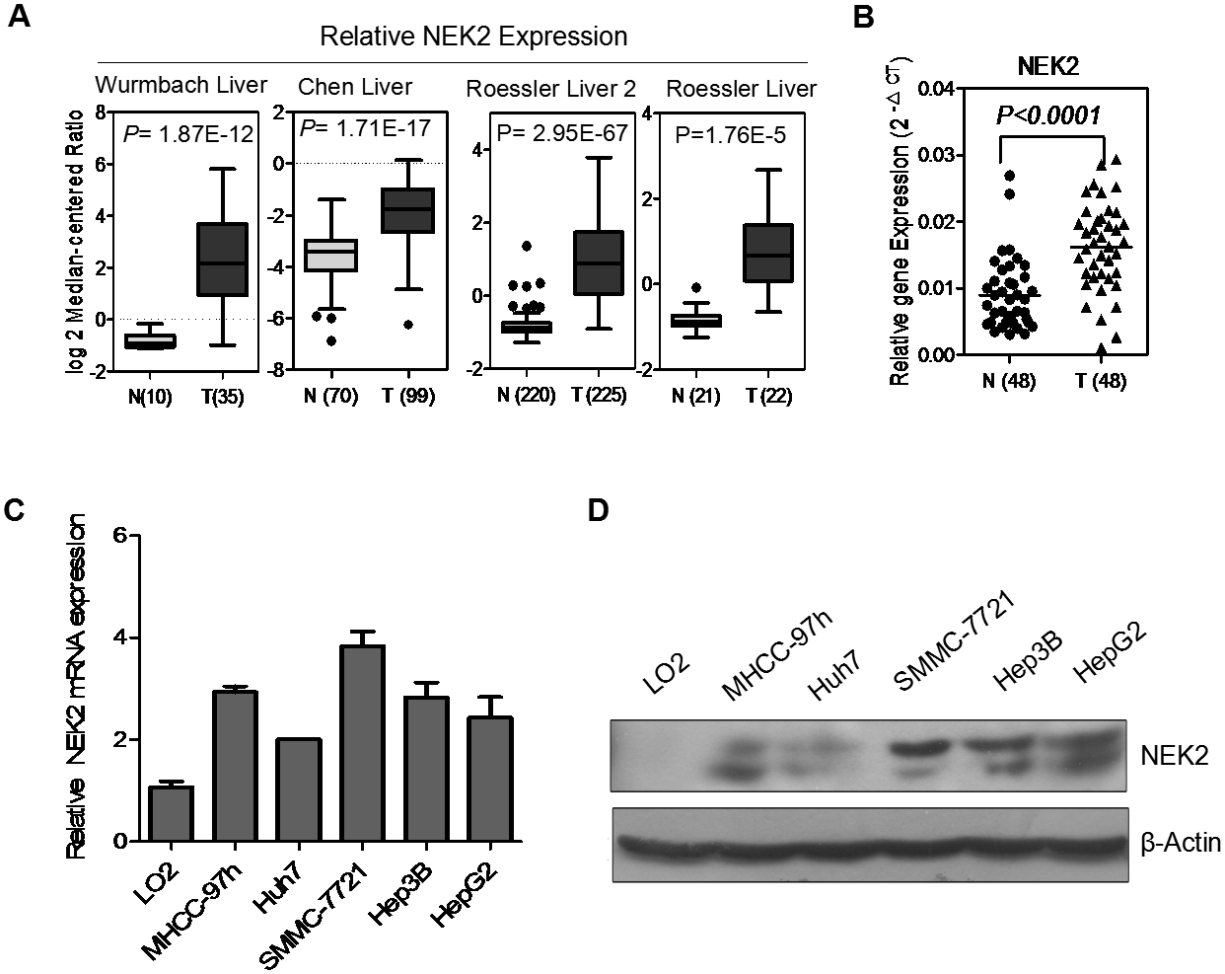
NEK2 expression in HCC tissues and cell lines **(A)** NEK2 mRNA is overexpressed in Wurmbach Liver, Chen liver, Roessler liver 2 and Roessler liver datasets from Oncomine database. **(B)** NEK2 mRNA expression is significantly higher in HCC tumor tissues compared to matched normal adjacent liver tissues. **(C)** qRT-PCR and **(D)** western blot. Both experiments show that the expression of NEK2 in all HCC cell lines, and the highest expression of NEK2 in SMMC-7721 cells and lowest expression in Huh7 cells.

First, we investigated the expression profile of NEK2 in human HCC in multiple patient datasets available in the Oncomine database, including Wurmbach Liver, Chen liver, Roessler liver 2 and Roessler liver and found that NEK2 was overexpressed in HCC patient samples compared to controls (Figure 1A). To validate this finding, we analyzed NEK2 mRNA levels in 48 paired HCC and normal adjacent liver tissues by qRT-PCR. We again observed that the NEK2 mRNA levels in the HCC tissues were significantly higher than the normal adjacent liver tissues (Figure 1B).

Next, we analyzed NEK2 expression in multiple HCC cell lines, MHCC-97H, SMMC-7721, Hep3B, HepG2, and Huh7 in comparison to the normal liver cell line LO2 by qRT-PCR and western blot. We observed that NEK2 was significantly upregulated in all the HCC cell lines compared with the normal liver cell line LO2 (Figure 1C, 1D). It was most highly in the SMMC-7721 cell line and was lowest in the Huh7 cell line (Figure 1C, 1D). Therefore, these two cell lines were selected for further experiments.

### Clinical significance of NEK2 expression in HCC patients

**Figure 2:**
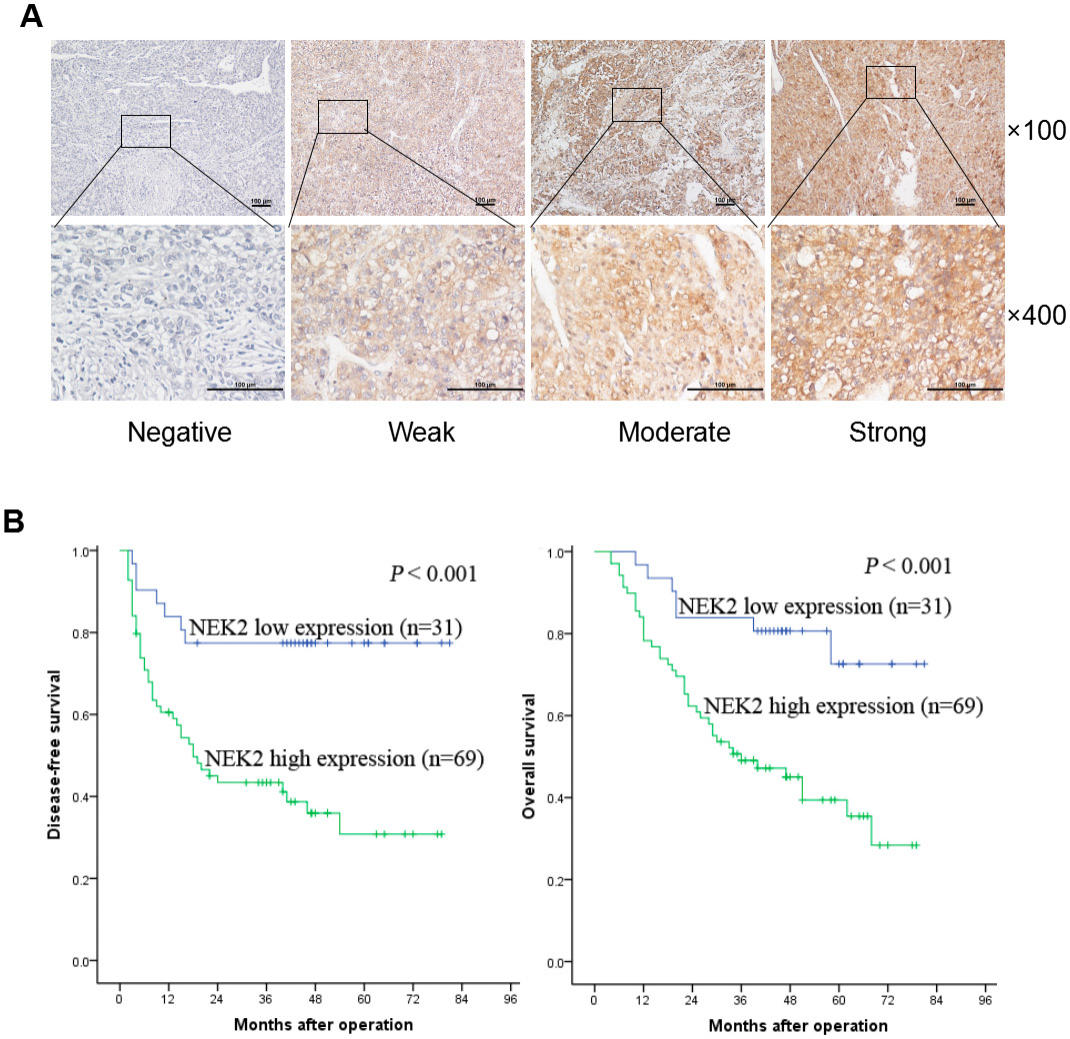
NEK2 immunohistochemical analysis in HCC patient tissues **(A)** Comparative analysis of cytoplasmic and membrane expression of NEK2 in the the HCC tissues is compared to adjacent normal tissues. The samples are scored as 0 (a, e), 1 + (b, f), 2 + (c, g), and 3 + (d, h) according to Shimizu criteria. The magnifications used are 100X (a-d) and 400X (e-h). **(B)** Kaplan–Meier survival curves show disease free survival (DFS) and overall survival (OS) for the NEK2 low expression group (scored as 0 and 1 +, n = 31) and the NEK2 high expression group (scored as 2 + and 3 +, n = 69) based on immunohistochemical analysis. The log-rank test shows that HCC patients with high NEK2 expression have lower disease-free survival (left) and overall survival (right) than those with low expression of NEK2.

Since we observed that NEK2 was constantly overexpressed in HCC tissues and cell lines, we then investigated the clinical significance of NEK2. Towards this, we examined NEK2 expression in 100 HCC patient tissue samples by immunohistochemical staining and observed that the patients could be classified into 2 categories based on the high or low expression of NEK2 (Figure 2A). High NEK2 expression was detected in 69% (69/100) of patient samples. The NEK2 expression levels significantly correlated with HBsAg, largest tumor size, Edmonson grading, macro- and micro-vascular invasion, Milan criteria, UCSF criteria, and Hangzhou criteria (Table 1). Kaplan-Meier survival analysis showed that the 1-, 3-, 5-year disease free survival (DFS) rates were 83.9%, 77.4%, 77.4% in patients with NEK2 low expression group compared to 60.5%, 43.4%, 30.8% in patients with NEK2 high expression group, respectively (*P* < 0.001; Figure 2B). Similarly, the 1-, 3-, 5-year overall survival (OS) rates were 96.8%, 83.9%, 72.6% in patients with NEK2 low expression group compared to 78.3%, 49.1%, 39.4% in patients with NEK2 high group (*P* < 0.001, Figure 2B). This was further corroborated by the multivariate Cox proportional hazard regression analysis that showed NEK2 expression was an independent prognostic factor for DFS and OS (Table 2 & Supplementary Table 2). Therefore, higher NEK2 expression suggested poor outcomes for HCC patients.

**Table 1:** Relationship between the expression of NEK2 and clinicopathological characteristics

Category	Subcategory	Cases	NEK2 expression		*P* value
			Low (n=31)	High (n=69)	
Gender	Male	94	27	67	
	Female	6	4	2	0.051
Age (years)	≤ 50	50	16	34	
	< 50	50	15	35	0.829
HBsAg	Positive	92	26	66	
	Negative	8	5	3	**0.045**
Child- pugh stage	A	63	22	41	
	B	32	9	23	
	C	5	0	5	0.244
Preoperative tumor therapy	Yes	44	13	31	
	No	56	18	38	0.555
AFP(ng/ml)	≤400	60	21	39	
	>400	40	10	30	0.289
Size of largest tumor (cm)	≤ 5	52	22	30	
	5 to 8	19	5	14	
	> 8	29	4	25	**0.026**
Tumor number	≤ 3	80	24	56	
	> 3	20	7	13	0.665
Edmonson grading	I-II	70	26	44	
	III-IV	30	5	25	**0.042**
Macro-vascular invasion	Yes	25	3	22	
	No	75	28	47	**0.018**
Micro-vascular invasion	Yes	19	2	17	
	No	81	29	52	**0.032**
Milan criteria	Within	46	20	26	
	Beyond	54	11	43	**0.013**
UCSF criteria	Within	56	22	34	
	Beyond	44	9	35	**0.043**
Hangzhou criteria	Within	59	23	36	
	Beyond	41	8	33	**0.038**

NEK2: NIMA (never in mitosis gene A)-related expressed kinase 2; HBsAg: hepatitis B surface antigen; AFP: alpha fetoprotein

**Table 2:** Prognostic factors for DFS and OS by multivariate Cox proportional hazards regression model

Variables	DFS			OS		
	HR	95%CI	*P*	HR	95%CI	*P*
Macro-vascular invasion	2.664	1.452-4.888	0.002	3.727	2.012-6.904	< 0.001
Size of largest tumor	2.082	1.477-2.934	< 0.001	1.725	1.233-2.415	0.001
NEK2 expression	3.371	1.492-7.618	0.003	3.082	1.373-6.918	0.006

HR: hazard ratio; CI: confidence interval. Other abbreviations as in Table 1.

### NEK2 promotes proliferation, colony formation, migration and invasion of HCC cell lines

**Figure 3:**
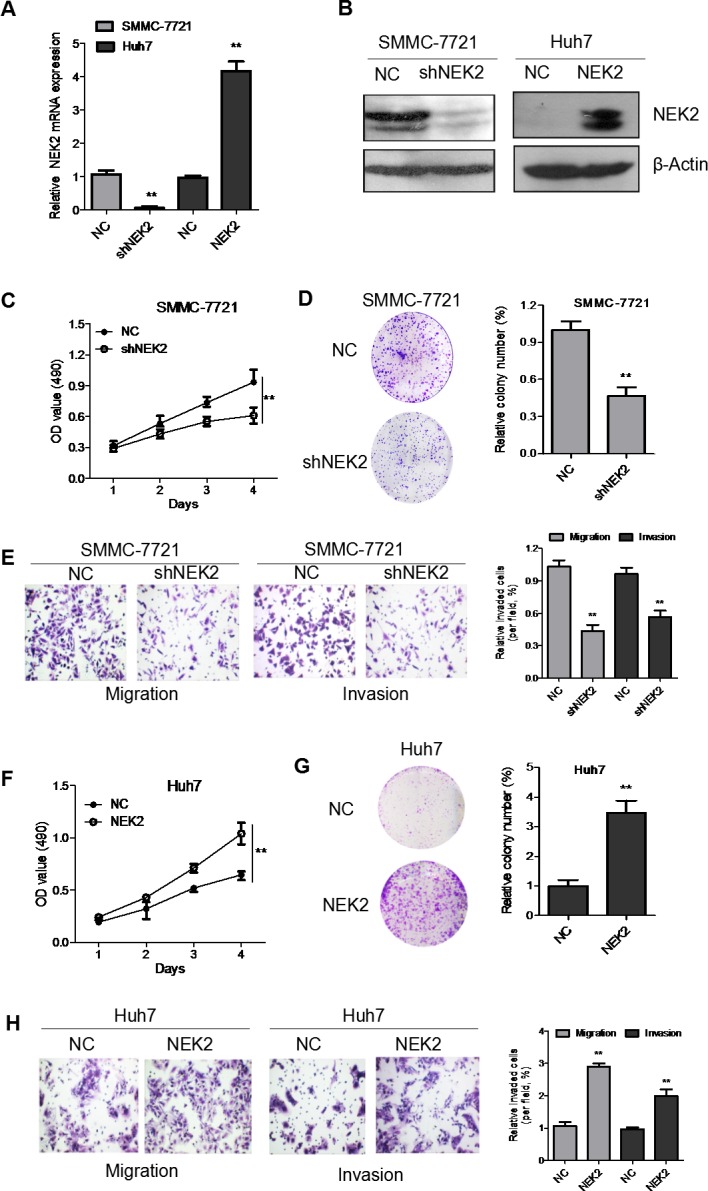
NEK2 accelerates proliferation, colony formation, migration and invasion of HCC cells *in vitro* **(A)** Quantitative real time PCR analysis of NEK2 expression in SMMC7721-NEK2shRNA cells and Huh7-NEK2 cells compared to controls. **(B)** Western blot analysis of NEK2 expression in SMMC7721-NEK2shRNA cells and Huh7-NEK2 cells compared to controls is shown. NEK2 shRNA downregulated NEK2 in the SMMC7721-NEK2shRNA cells compared to the controls; NEK2 overexpression plasmid upregulated NEK2 by in Huh7-NEK2 cells. **(C)** MTT assays show that NEK2 downregulation decreased cell proliferation in SMMC7721-NEK2 shRNA cells. **(D)** Colony formation assays indicate significantly decreased the number of colonies in the SMMC7721-NEK2shRNA cells compared to control SMMC7721 cells. **(E)** Transwell migration and invasion of SMMC7721-NEK2 shRNA cells were significantly lower than the control SMMC7721 cells. **(F)** MTT assays show that Huh7-NEK2 cell demonstrated enhanced cell proliferation compared to the control Huh7 cells. **(G)** Colony formation assays indicate the Huh7-NEK2 cells formed higher number of colonies compared to control Huh7 (NC) cells. **(H)** Transwell migration and invasion of Huh7-NEK2 cells is significantly elevated compared with control Huh7 cells. All experiments were repeated thrice. *P < 0.05, compared to control.

To understand the role of NEK2 in HCC progression, we transfected Huh7 cells with a NEK2 overexpression plasmid to enhance NEK2 levels in the low-expressing Huh7 cell line. Concurrently, we transfected NEK2 shRNA to inhibit NEK2 expression in high expressing SMMC-7721 cell line. Analysis by qRT-PCR and western blot demonstrated that NEK2 expression was significantly upregulated in Huh7 cells transfected with the NEK2 overexpression plasmid (Figure 3A, 3B). Similarly, NEK2 shRNA effectively inhibited NEK2 expression in SMMC-7721 cells (Figure 3A, 3B). We used the stable transfectants to further study the role of NEK2 in HCC progression.

To investigate the biological functions of NEK2 in HCC proliferation, we performed *in vitro* proliferation assays. As shown in Figure 3C, there was significant decrease in cell proliferation in SMMC7721 cells transfected by the NEK2 shRNA (SMMC-7721-shNEK2). Conversely, when NEK2 was overexpressed in Huh7 cells (Huh7-NEK2), cell proliferation was significantly enhanced (Figure 3F). Furthermore, SMMC-7721-shNEK2 cells formed significantly fewer colonies compared to the control SMMC-7721-NC cells in the colony formation assays (Figure 3D). Similarly, Huh7-NEK2 cells formed greater number of colonies than the control Huh7 cells (Figure 3G). These data suggested that higher NEK2 levels promoted proliferation of HCC cells.

Next, we performed transwell migration and invasion assays to test if NEK expression modulates the cell migration and invasiveness. We observed that the SMMC-7721-shNEK2 cells were less migratory and invasive than the control SMMC-7721 cells (Figure 3E). Conversely, Huh7-NEK2 cells were more migratory and invasive than the control Huh7 cells (Figure 3H). Together, these results showed that higher NEK2 levels promoted proliferation, colony formation, migration and invasion of HCC cells *in vitro* and implied a probable role for NEK2 in HCC progression and metastasis.

### NEK2 promotes HCC tumor growth *in vivo*

**Figure 4:**
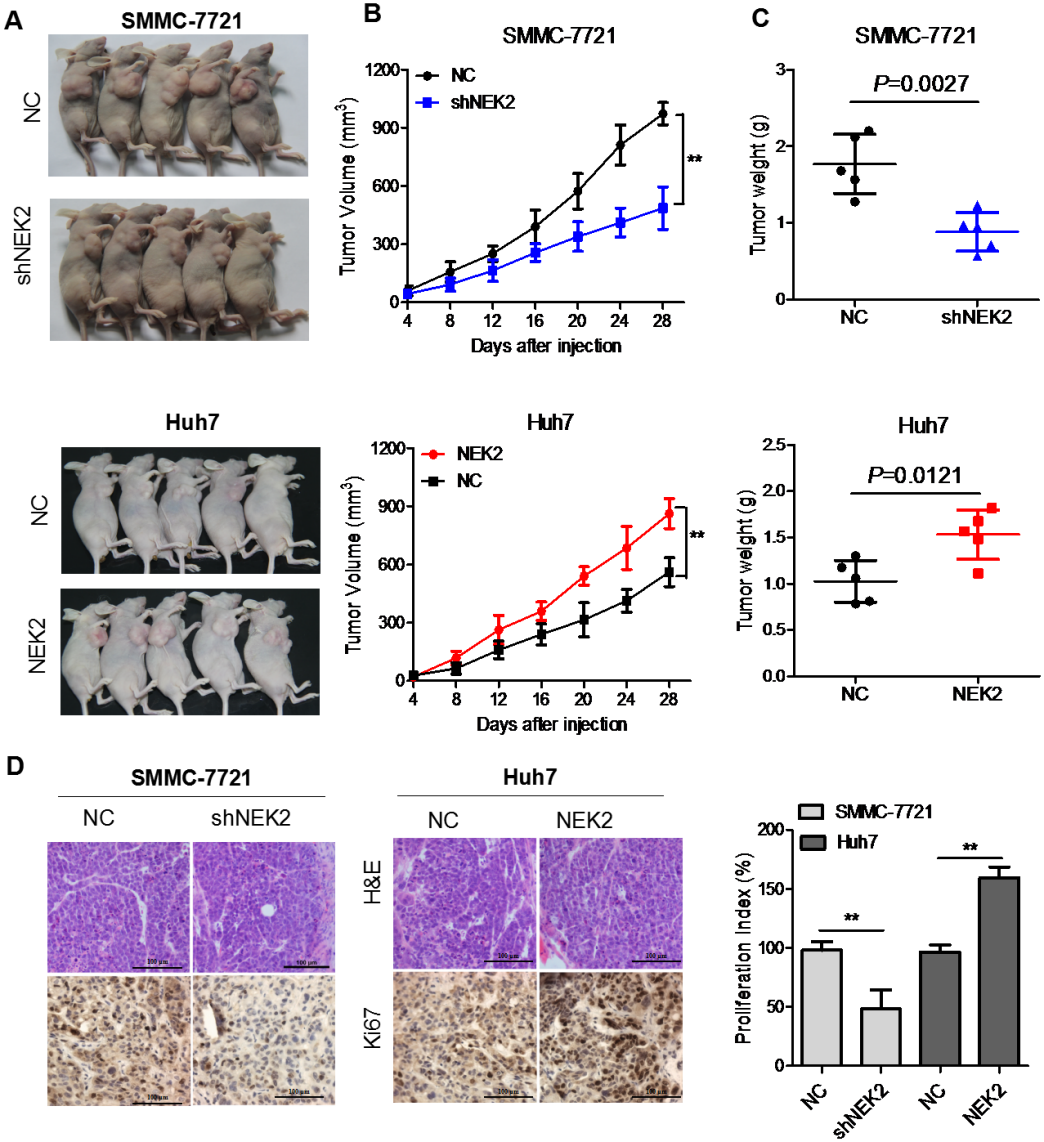
NEK2 promotes HCC tumor growth *in vivo* **(A)** Representative images of day 28 tumors in mice transplanted with SMCC7721-shNEK2, SMCC7721 (control), Huh7-NEK2 and Huh7 (control) cells. **(B)** Plots showing tumor growth measurements of SMCC7721-shNEK2, SMCC7721 (control), Huh7-NEK2 and Huh7 (control) cells is shown. **(C)** The mean tumor weights in each group (SMCC7721-shNEK2, SMCC7721 (control), Huh7-NEK2 and Huh7 (control)) on day 28 is shown. **(D)** IHC staining showing that cell proliferation (Ki67-positive) positively correlates with NEK2 expression levels. Data in **(B)** and **(C)** are presented as mean ± SD (n=5). *P < 0.05 versus control.

To investigate the biological significance of NEK2 in HCC, we injected SMCC-7721-shNEK2 and Huh7-NEK2 cells with their corresponding controls subcutaneously into nude mice and monitored the tumor growth. The SMCC-7721-shNEK2 cells demonstrated significantly reduced size tumors than the control SMCC-7721 cells (Figure 4A). Conversely, Huh7-NEK2 cells formed significantly larger tumors than the Huh7 control cells (Figure 4A). Furthermore, high NEK2 levels in the HCC cells corresponded to greater tumor growth rate and tumor weight (Figure 4B, 4C). IHC staining showed that tumors formed from cells with higher NEK2 were more Ki67-positive (cell proliferation index) (Figure 4D). Collectively, these data suggested that NEK2 promoted HCC tumor growth *in vivo*.

### MiR-486-5p targets NEK2

**Figure 5:**
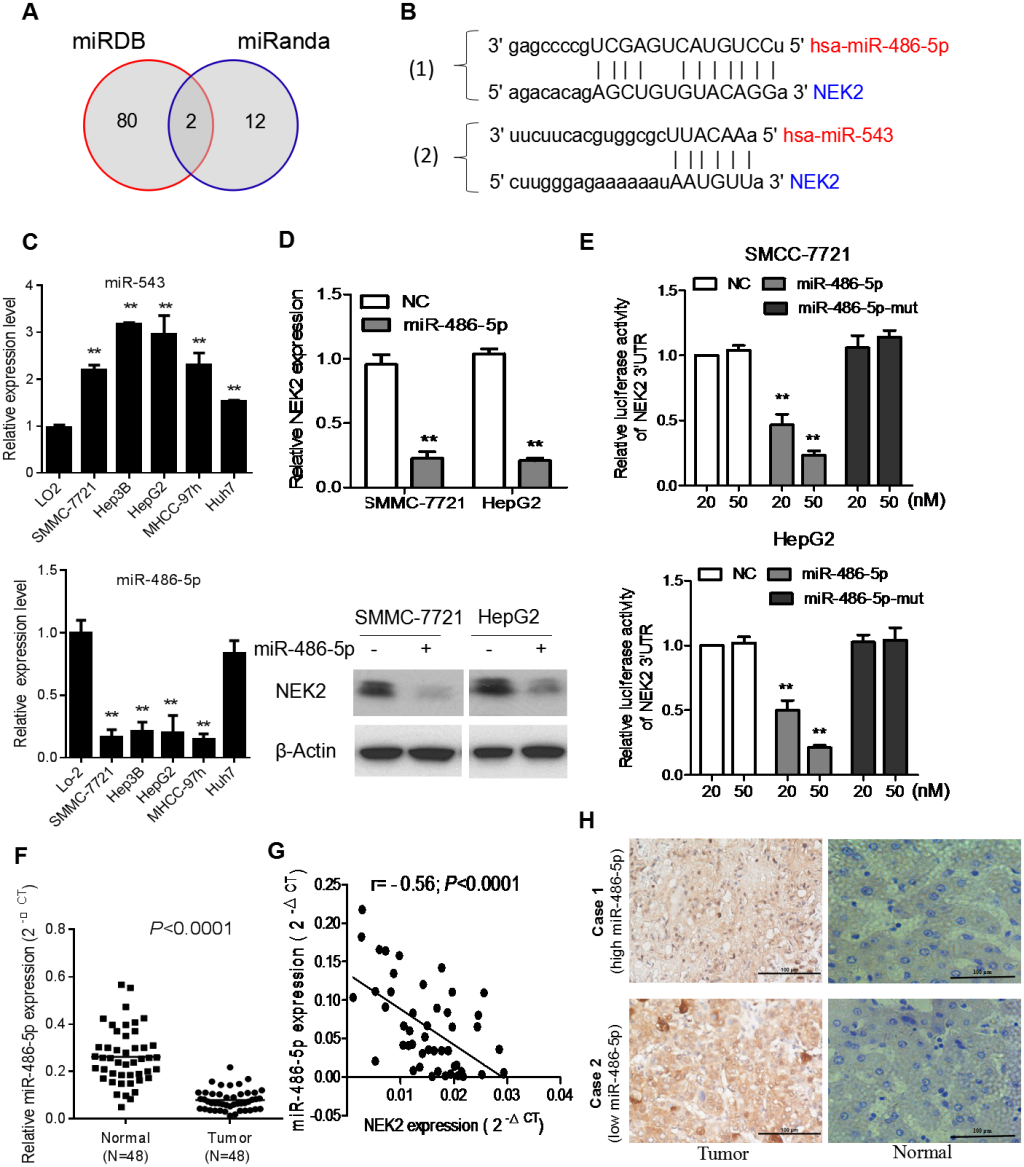
NEK2 is a target for miR-486-5p in HCC cells **(A)** Venn diagrams showing the number of potential miRNAs targeting the 3′UTR of NEK2, as predicted by two databases, miRanda and miRDB. **(B)** Sequences of miR-486-5p and miR-543 and their potential binding sites in the 3′UTR of NEK2 is shown. **(C)** Quantitative real time PCR analyzing miR-486-5p expression relative to U6 as internal control is shown. **(D)** Comparison of NEK2 expression in HCC cells transfected with miR-486-5p mimic or negative control (NC) based on qRT-PCR and western blotting. The loading control for western blotting was β-Actin. **(E)** Analysis of luciferase activity from reporters containing the 3′UTR end of NEK2 in cells transfected with the miR-486-5p mimic, miR-486-5p mutation mimic (miR-486-5p-mut) and negative control (NC) is shown. **(F)** The expression levels of miR-486-5p in 48 pairs of human HCC tissues (T) and adjacent normal liver tissues (N). **(G)** Correlation analysis of miR-486-5p expression with NEK2 mRNA expression in HCC patient samples (n=48). **(H)** Representative IHC staining showing the inverse association between miR-486-5p expression and NEK2 levels in human HCC specimens. Two representative cases are shown (scale bar: 100μm). Means ± SD (n=3) are shown in (C, D and E). *P < 0.05 versus control cells.

Finally, we investigated the mechanism that regulates NEK2 expression. Since microRNAs are master regulators of gene expression and play important role in tumorigenesis, we sought to identify miRNAs that regulate NEK2. Therefore, we analyzed mRNA target-predicting algorithms, miRanda and miRDB to identify potential miRNAs that bind to 3′ UTR of NEK2 and identified miR-543 and miR-486-5p as possible candidates (Figure 5A, 5B). Next, we analyzed the expression levels of miR-543 and miR-486-5p in normal liver cell LO2 and HCC cell lines, namely, SMMC-7721, Hep3B and HepG2 by qRT-PCR. In general, miR-543 was upregulated and miR-486-5p was downregulated in the HCC cells compared to the LO2 cells (Figure 5C & Supplementary Figure 1). Therefore, we postulated that miR-486-5p was potential critical upstream negative regulator of NEK2 that maybe relevant for cancer therapy.

To establish the role of miR-486-5p in regulating NEK2 expression, we transfected HCC cells with miR-486-5p or mutant miR-486-5p mimics and luciferase tagged NEK2-3′ UTR and analyzed the effects after 48h by luciferase reporter assay. We observed that the miR-486-5p mimics attenuated luciferase activity of NEK2-3′ UTR whereas the mutant miR-486-5p mimics did not suppress NEK2-3′ UTR luciferase activity (Figure 5E). Further, we analyzed the 48 matched pairs of HCC and normal adjacent liver tissue samples by qRT-PCR to determine miR486-5p expression and found it to be significantly downregulated in HCC tissues (Figure 5F). Also, there was an inverse correlation between the expression of miR-486-5p and NEK2 in HCC tissues (Figure 5G). IHC analysis revealed that NEK2 levels were low in high miR-486-5p expressing tissues and conversely tissues with high NEK2 had diminished levels of miR-486-5p (Figure 5H). Therefore, these results demonstrated that NEK2 was regulated by miR-486-5p and is potential consequential for HCC patient outcomes.

## DISCUSSION

NIMA (never in mitosis A) protein was first described by Ron Morris in 1975 [23]. The NIMA related kinase family consists of 11 members in mammals, of which NEK2 shares the greatest sequence similarity to NIMA [6]. NEK2 regulates centrosome separation and spindle formation [24]. NEK2 overexpression results in centrosomal abnormalities and leads to aneuploidy karyotype by disrupting the mitotic checkpoint [10, 11, 24, 25]. In addition, overexpression of NEK2 has been demonstrated in many tumors and associated with aggressive cancer phenotype and poor prognosis in pancreatic ductal adenocarcinoma, colorectal carcinoma, cholangiocarcinoma, and breast cancer. Zhang et al. first reported that NEK2 was upregulated in HCC tissues compared to adjacent tissues and promoted *in vitro* proliferation, cell cycle, migration and invasion of HepG2 cells [20]. Lin et al. reported that NEK2 was associated with unfavorable outcomes in HCC patients after liver resection and regulated self-renewal and chemotherapeutic resistance in HCC cells [21]. Recently, Wubetu et al. showed that high NEK2 expression was a predictor of tumor recurrence in hepatocellular carcinoma patients after hepatectomy [22]. However, systematic analysis of its role in HCC progression is lacking. The upstream regulators of NEK2 are not clear and its prognostic role in hepatocellular carcinoma patients after liver transplantation is unclear.

Hence, we systemically investigated the role of NEK2 in HCC progression in this study. Firstly, our analysis revealed that NEK2 was overexpressed in HCC patient tissues and cell lines. Secondly, we confirmed that NEK2 was an independent prognostic marker for HCC patients after liver transplant. Further, we confirmed that high NEK2 expression promoted cell proliferation, colony formation, migration and invasion *in vitro* and tumor growth *in vivo*.

Previously, NEK2 was identified as the target gene for cancerous inhibitor of protein phosphatase 2A (CIP2A) induced tumorigenesis [26]. Also, silencing of cyclin dependent kinase 4 (CDK4) in Her2^+^ breast cancer cells resulted in diminished levels of NEK2 [27]. In this study, we identified that miR-486-5p directly regulated NEK2 by binding to its 3′ UTR. MicroRNAs are small non-coding RNAs (19-22 nucleotides) that are master regulators of gene expression and play a critical role in tumorigenesis [28]. Aberrant expression of miR-486-5p is associated with different types of diseases. Inhibition of miR-486-5p in colorectal carcinoma cells enhances tumor growth and lymphangiogenesis [29]. Also, miR-486-5p is significantly downregulated in non-small-cell lung cancer (NSCLC) and NSCLC cell lines resulting in tumor progression and metastasis [30]. Huang et al. demonstrated that miR-486-5p was downregulated in HCC; miR-486-5p suppressed HCC growth, migration and invasion by targeting PIK3R1 [31]. Hence, we validated definitively that NEK2 was a direct target of miR-486-5p. Therefore, enforced expression of miR-486-5p can be a potential therapeutic strategy for HCC patients with NEK2 high expression and needs to be explored further.

In summary, our study demonstrates that NEK2 is frequently overexpressed in HCC and is associated with aggressive HCC tumor phenotype. High NEK2 expression also indicates poor prognosis for HCC patients after liver transplant. Finally, we identified miR-486-5p as an upstream regulator of NEK2 expression, thereby potentially opening up a future therapeutic avenue to alleviate HCC progression.

## MATERIALS AND METHODS

### NEK2 gene expression data in HCC patients in the oncomine database

The Oncominemicroarray database (http://www.oncomine.org) was used to screen the gene expression level in HCC samples compared with normal liver tissues. *NEK2* was used as the keyword in the Oncomine query; Cancer vs. Normal Analysis was used as the primary filter; and Liver cancer was chosen as the cancer type. The NEK2 gene expression data were log-transformed, median-centered in each array, and the standard deviation (SD) was normalized to one in each array [32].

### HCC patient data and tissue specimens

We obtained 100 HCC tissue specimens from patients who underwent liver transplantation at the Organ Transplant Center of the First Affiliated Hospital, Sun Yat-sen University (Guangzhou, China) from January 2008 to July 2013. Further forty-eight matched pairs of HCC and normal adjacent liver tissue samples were acquired during surgery from June 2014 to April 2015 (Supplementary Table 1). The samples were snap-frozen in liquid nitrogen and stored at −80°C for later experiments including RNA extraction or formalin fixation and paraffin embedding for immunohistochemistry. Disease-free survival (DFS) was defined as the length of time after liver transplantation during which a patient survived without any signs of HCC or the last follow-up. Overall survival (OS) was defined as the length of time the patient survived after the date of operation or until the last follow-up date. The patients’ follow up was performed as previously described [33, 34]. The last follow-up date was October 31^st^, 2015. The complete clinical and pathological features of these patients were collected and stored in our database. The study protocol followed the Ethical Guidelines of the 1975 Declaration of Helsinki, which were revised in 2000. All patients gave written informed consent on the use of clinical specimens for medical research. Researches on human materials were approved by the Ethics committee of the First Affiliated Hospital of Sun Yat-sen University.

### HCC cell lines and cell culture

The human HCC cell line HepG2 was purchased from the American Type Culture Collection (ATCC; Rockville, MD, USA). The human HCC cell lines MHCC-97H, Huh7, SMMC-7721, Hep3B, and normal liver cells LO2 were all obtained from the institute of Biochemistry and Cell Biology, Chinese Academy of Sciences, Shanghai, China and validated.

The cell lines were grown in low glucose Dulbecco's modified Eagle media (DMEM), containing 10 % fetal bovine serum (FBS) supplemented with 100 U/ml penicillin and 0.1 mg/ml streptomycin at 37°C and 5% CO_2_.

### NEK2 immunohistochemical analysis

Immunohistochemistry (IHC) was performed as previously described [35] using the NEK2 antibody (#sc-33167) that was purchased from Santa Cruz Biotechnology (Santa Cruz, USA). Immunohistochemical analysis was performed by two independent investigators who were blinded to the clinical outcome. The NEK2 protein expression in HCC specimens was scored as 0 to 3+ using the Shimizu criteria [36]. The expression levels of NEK2 protein were divided into low expression (0 or 1+) and high expression (2+ or 3+) groups.

### Generation of high and low NEK2 expressing HCC cell lines

NEK2 RNA interference (RNAi) plasmid and NEK2 overexpression plasmid were obtained from Forevergen Biosciences (Guangzhou, China). The Oligo sequence for NEK2 shRNA was 5′-AACTTTCTGAGAGTCAGCTCACATTCAAGAGATG TGAGCTGACTCTCAGAATTTTTTCTCGAG-3′. SMMC-7721 cells were transfected with NEK2 shRNA or scramble shRNA; Huh7 cells were transfected with NEK2 overexpression plasmid or vector control, as previously described [37]. The transfected cells were selected with 0.5mg/mL puromycin. Stably transfected clones were validated by qRT-PCR and western blot analysis.

### Quantitative real time polymerase chain reaction and western blotting

Quantitative real time polymerase chain reaction (qRT-PCR) and western blotting (WB) were performed as previously described [38]. Transcripts for NEK2 and β-actin (control) were analyzed by qRT-PCR. The following primers were used for qRT-PCR: NEK2-F: 5′-ATCTCTAGAATGCCTTCCCGGGCTGAG3′; NEK2-R: 5′-ATACGGATCCCTAGCGCATGCCCAGGATC3′; β-actin-F: 5′-TCACCAACTGGGACGACAT-3′; β-actin-R: 5′-GCACAGCCTGGATAGCAAC-3′.

### Cell proliferation assay

The proliferation of HCC cells was measured using MTT assay. Logarithmically growing HCC cell lines (five repeat wells per group) were inoculated into 96-well culture plates at a density of 5×10^3^ cells/well. Then, 100μl fresh DMEM medium with 0.5mg/ml MTT was added into each well on days 1-4 and incubated at 37°C for 4 hours followed by replacing the medium with 100μl DMSO and shaken gently at room temperature for 10 minutes. The absorbance was measured at 490 nm.

### Colony formation assay

For colony formation assay, 500 cells of each cell type were seeded into six-well plate and gently shaken and incubated at 37°C and 5% CO_2_ for 2 weeks. Subsequently, the medium was removed and the cells stained with 0.1% crystal violet (Beyotime Institute of Biotechnology, Shanghai, China) followed by counting positive colonies (diameter > 40 μm) after imaging. The differences in the colony formation ability of different cell types were documented.

### Transwell migration and invasion assay

For the transwell migration assay, 5×10^4^ cells in serum free DMEM medium were seeded into the upper chamber of 8μm transwell inserts (BD Biosciences, Franklin Lakes, NJ) and DMEM with 10% bovine serum albumin (BSA) was added in the lower chamber. After incubation for 24h at 37°C, cells in the upper chamber were removed carefully and the cells adhering to the underside of the transwell membrane were fixed in 20% methanol and stained with 0.1% crystal violet. The number of cells was then counted under an inverted microscope (Nikon, Chiyoda-Ku, Japan). For the transwell invasion assay, all procedures were the same as the transwell migration assay, except that the upper chamber was coated with matrigel (BD Biosciences, Franklin Lakes, NJ).

### Luciferase reporter assay

DNA fragments from the 3′-UTR of NEK2 that contained the predicted complementary sites of miR-486-5p were cloned into a pGL3-basic vector (Addgene, Cambridge, USA). MiR-486-5p and mutant miR-486-5p mimics were purchased from RiboBio (Guangzhou, China). The HCC cells (10,000/well) were seeded in triplicate in 48 well plates and allowed to settle for 24h. Then, the pGL3-NEK2-3′UTR reporter plasmids (100ng) plus 5ng of pRL-TK renilla plasmid (Promega, Madison, USA) and increasing levels (10nM and 50 nM) of negative control (NC), miR-486-5p or mutant miR-486-5p mimics were co-transfected into the HCC cells using the Lipofectamine LTX reagent (Invitrogen, Carlsbad, USA) according to the manufacturer's instructions. The luciferase and renilla signals were measured 24h after transfection using the Dual Luciferase Reporter Assay Kit (Promega, Madison, USA) according to the protocol provided by the manufacturer.

### *In vivo* xenograft tumor growth

For the xenograft tumor growth assay, HCC cells (1×10^6^) were injected subcutaneously into the right flank of 5 week-old male BALB/C nude mice (5 mice per group). Tumors were monitored for 28days and the tumor volume was measured every 4 days and calculated by the formula V = 0.5 × L ×W^2^. All animal experiments were performed as approved by the Committee on the Use of Live Animals in Teaching and Research at the First Affiliated Hospital of Sun Yat-sen University.

### Statistical analysis

Statistical analysis was performed with the SPSS software (19.0; SPSS, Inc., Chicago, IL). Values are expressed as mean ± standard deviation (SD). The Student's *t* test was used for comparisons between groups. Categorical data were analyzed by the chi-square or Fisher's exact tests. Cumulative recurrence and survival rates were analyzed using Kaplan-Meier's method and the log-rank test. Factors identified as significant (*P* < 0.1) on univariate analysis were further analyzed by the multivariate competing risk Cox regression model to identify significant independent predictors of HCC recurrence and overall survival. The final multivariate model was performed using the forward stepwise procedure for variable selection. *P* < 0.05 was considered statistically significant.

## SUPPLEMENTARY MATERIALS FIGURES AND TABLES




