# Supplementary material for: MiR-486-5p negatively regulates oncogenic NEK2 in hepatocellular carcinoma
Source: Oncotarget. 2017 May 5;8(32):52948–59. doi: 10.18632/oncotarget.17635 (PMC5581084; doi:10.18632/oncotarget.17635)
Supplement: Supplementary file 1 [file oncotarget-08-52948-s001.pdf]

MiR-486-5p negatively regulates oncogenic NEK2 in hepatocellular carcinoma

SUPPLEMENTARY MATERIALS

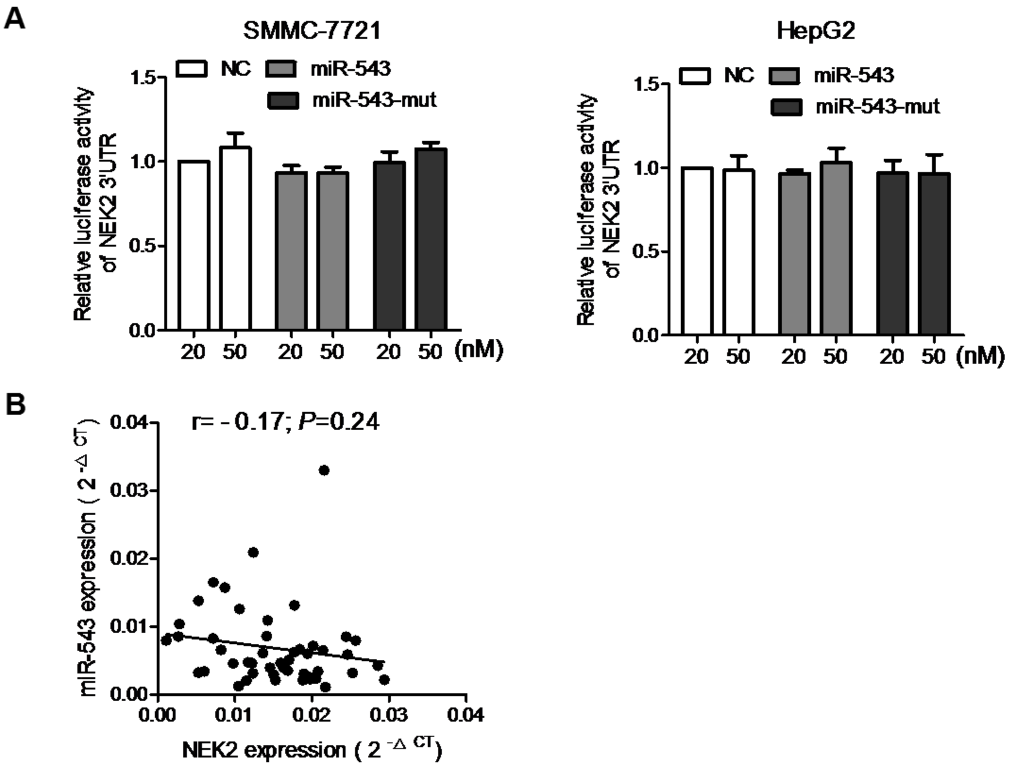

Supplementary Figure 1: (A) Analysis of luciferase activity from reporter constructs containing the 3'UTR end of NEK2 in HCC cells transfected with the miR-543 mimic, miR-543 mutation mimic (miR-543-mut) and negative control (NC). **(B)** Correlation analysis of miR-543 expression with NEK2 mRNA expression in HCC patient samples (n=48).

Supplementary Table 1: Clinicopathological characteristics of 48 HCC patients

See Supplementary File 1

Supplementary Table 2: Influence of clinicopathological characteristics on patients' prognosis

See Supplementary File 1
