# Supplementary material for: MiR-486-5p negatively regulates oncogenic NEK2 in hepatocellular carcinoma
Source: Oncotarget. 2017 May 5;8(32):52948–59. doi: 10.18632/oncotarget.17635 (PMC5581084; doi:10.18632/oncotarget.17635)
Supplement: Supplementary file 2 [file oncotarget-08-52948-s002.doc]

**Supplementary Table 1. Clinicopathological characteristics** of 48 HCC patients

| **Category** | **Subcategory** | **Cases** |
| --- | --- | --- |
|  |  | n=48 |
| Gender | Male | 44 |
|  | Female | 4 |
| Age (years) | ≤ 50 | 22 |
|  | < 50 | 26 |
| HBsAg | Positive | 46 |
|  | Negative | 2 |
| Child- pugh stage | A | 30 |
|  | B | 16 |
|  | C | 2 |
| Preoperative tumor therapy | Yes | 22 |
|  | No | 26 |
| AFP(ng/ml) | ≤400 | 29 |
|  | >400 | 19 |
| Size of largest tumor (cm) | ≤ 5 | 25 |
|  | 5 to 8 | 10 |
|  | > 8 | 13 |
| Tumor number | ≤ 3 | 38 |
|  | > 3 | 10 |
| Edmonson grading | I-II | 34 |
|  | III-IV | 14 |
| Macro-vascular invasion | Yes | 10 |
|  | No | 38 |
| Micro-vascular invasion | Yes | 9 |
|  | No | 39 |
| Milan criteria | Within | 22 |
|  | Beyond | 26 |
| UCSF criteria | Within | 27 |
|  | Beyond | 21 |
| Hangzhou criteria | Within | 30 |
|  | Beyond | 18 |

The abbreviations as in Table 1.

**Supplementary Table 2. Influence of clinicopathological** characteristics on patients’ prognosis

| **Variables** | **n** | **DFS** | | | | **OS** | | |  |
| --- | --- | --- | --- | --- | --- | --- | --- | --- | --- |
|  |  | 1-yr | 3-yr | 5-yr | *P* | 1-yr | *3*-yr | 5-yrs | *P* |
| Gender |  |  |  |  |  |  |  |  |  |
| Male | 94 | 65.7% | 52.3% | 44.6% |  | 83.0% | 57.4% | 46.5% |  |
| Female | 6 | 100.0% | 88.3% | 88.3% | 0.116 | 100.0% | 100.0% | 100.0% | 0.125 |
| Age (years) |  |  |  |  |  |  |  |  |  |
| ≤ 50 | 50 | 60.0% | 47.9% | 43.6% |  | 82.0% | 56.0% | 45.1% |  |
| > 50 | 50 | 75.8% | 60.5% | 50.6% | 0.187 | 86.0% | 63.9% | 54.5% | 0.439 |
| HBsAg |  |  |  |  |  |  |  |  |  |
| Positive | 92 | 66.1% | 53.7% | 46.0% |  | 83.7% | 58.6% | 48.7% |  |
| Negative | 8 | 87.5% | 58.3% | 58.3% | 0.517 | 87.5% | 75.0% | 62.5% | 0.532 |
| Child-pugh stage |  |  |  |  |  |  |  |  |  |
| A | 63 | 61.6% | 46.2% | 38.4% |  | 82.5% | 57.1% | 50.1% |  |
| B | 32 | 75.0% | 62.5% | 57.7% |  | 84.4% | 59.4% | 39.9% |  |
| C | 5 | 100.0% | 100.0% | 100.0% | 0.047 | 100.0% | 100.0% | 100.0% | 0.224 |
| Preoperative tumor therapy |  |  |  |  |  |  |  |  |  |
| Yes | 44 | 68.2% | 49.4% | 35.0% |  | 88.6% | 61.4% | 42.9% |  |
| No | 56 | 67.6% | 58.1% | 52.8% | 0.452 | 80.4% | 58.8% | 53.6% | 0.803 |
| AFP (ng/ml) |  |  |  |  |  |  |  |  |  |
| ≤400 | 60 | 81.7% | 71.5% | 61.6% |  | 96.7% | 78.3% | 61.6% |  |
| >400 | 40 | 46.6% | 27.1% | 22.6% | < 0.001 | 65.0% | 32.5% | 29.8% | < 0.001 |
| Size of largest tumor (cm) |  |  |  |  |  |  |  |  |  |
| ≤ 5 | 52 | 84.7% | 72.8% | 68.3% |  | 98.3% | 79.6% | 64.5% |  |
| 5 to 8 | 19 | 62.5% | 41.7% | 27.8% |  | 81.3% | 50.0% | 41.7% |  |
| > 8 | 29 | 30.2% | 15.1% | 10.1% | < 0.001 | 52.0% | 20.0% | 20.0% | < 0.001 |
| Tumor number |  |  |  |  |  |  |  |  |  |
| ≤ 3 | 80 | 72.5% | 60.8% | 51.1% |  | 82.5% | 63.6% | 50.0% |  |
| > 3 | 20 | 48.0% | 26.7% | 26.7% | 0.020 | 90.0% | 45.0% | 45.0% | 0.258 |
| Edmondson grading |  |  |  |  |  |  |  |  |  |
| I-II | 70 | 75.0% | 61.1% | 56.5% |  | 88.2% | 69.0% | 56.8% |  |
| III-IV | 30 | 52.0% | 39.0% | 29.5% | 0.017 | 75.0% | 40.6% | 34.8% | 0.015 |
| Macro-vascular invasion |  |  |  |  |  |  |  |  |  |
| Yes | 25 | 45.1% | 12.0% | 6.0% |  | 67.9% | 14.3% | 14.3% |  |
| No | 75 | 76.4% | 69.2% | 61.0% | < 0.001 | 90.3% | 77.7% | 63.1% | < 0.001 |
| Micro-vascular invasion |  |  |  |  |  |  |  |  |  |
| Yes | 19 | 50.0% | 10.0% | 0 |  | 75.0% | 16.7% | 0 |  |
| No | 81 | 70.3% | 59.6% | 53.0% | < 0.001 | 85.2% | 65.8% | 56.5% | < 0.001 |
| NEK2 expression |  |  |  |  |  |  |  |  |  |
| Low | 31 | 83.9% | 77.4% | 77.4% |  | 96.8% | 83.9% | 72.6% |  |
| High | 69 | 60.5% | 43.4% | 30.8% | < 0.001 | 78.3% | 49.1% | 39.4% | 0.001 |
| Milan criteria |  |  |  |  |  |  |  |  |  |
| Within | 46 | 87.8% | 79.5% | 76.8% |  | 98.0% | 89.8% | 71.0% |  |
| Beyond | 54 | 46.4% | 28.5% | 18.5% | < 0.001 | 70.6% | 31.4% | 29.1% | < 0.001 |
| UCSF criteria |  |  |  |  |  |  |  |  |  |
| Within | 56 | 86.0% | 77.0% | 72.4% |  | 94.7% | 85.9% | 67.3% |  |
| Beyond | 44 | 43.3% | 22.6% | 14.6% | < 0.001 | 69.8% | 25.6% | 25.6% | < 0.001 |
| Hangzhou criteria |  |  |  |  |  |  |  |  |  |
| Within | 59 | 84.4% | 73.2% | 69.1% |  | 93.8% | 82.8% | 65.8% |  |
| Beyond | 41 | 37.7% | 18.8% | 10.0% | < 0.001 | 66.7% | 19.4% | 19.4% | < 0.001 |

DFS: disease-free survival; OS: overall survival. Other abbreviations as in Table 1
